# Quantifying the pattern of retinal vascular orientation in diabetic retinopathy using optical coherence tomography angiography

**DOI:** 10.1038/s41598-021-95219-9

**Published:** 2021-08-04

**Authors:** Yanhui Ma, Matthew P. Ohr, Xueliang Pan, Cynthia J. Roberts

**Affiliations:** 1 Department of Ophthalmology and Visual Sciences, The Ohio State University, Columbus, OH USA; 2 Department of Biomedical Informatics, The Ohio State University, Columbus, OH USA; 3 Department of Biomedical Engineering, The Ohio State University, Columbus, OH USA

**Keywords:** Eye diseases, Biomedical engineering, Medical imaging

## Abstract

Quantitative imaging using optical coherence tomography angiography (OCTA) could provide objective tools for the detection and characterization of diabetic retinopathy (DR). In this study, an operator combining the second derivative and Gaussian multiscale convolution is applied to identify the retinal orientation at each pixel in the OCTA image. We quantified the pattern of retinal vascular orientation and developed three novel quantitative metrics including vessel preferred orientation, vessel anisotropy, and vessel area. Each of eight 45º sectors of the circular disk centered at the macular region was defined as the region of interest. Significant sectoral differences were observed in the preferred orientation (p < 0.0001) and vessel area (p < 0.0001) in the 34 healthy subjects, whereas vessel anisotropy did not demonstrate a significant difference among the eight sectors (p = 0.054). Differential retinal microvascular orientation patterns were observed between healthy controls (n = 34) and the DR subjects (n = 7). The vessel area characterized from the vascular orientation pattern was shown to be strongly correlated with the traditionally reported vessel density (Pearson R > 0.97, p < 0.0001). With three metrics calculated from the vascular orientation pattern simultaneously and sectorally, our quantitative assessment for retinal microvasculature provides more information than vessel density alone and thereby may enhance the detection of DR. These preliminary results suggest the feasibility and advantage of our vessel orientation-based quantitative approach using OCTA to characterize DR-associated changes in retinal microvasculature.

## Introduction

Diabetic retinopathy (DR) is the leading cause of vision impairment and blindness among working-age adults in the United States and worldwide^1,2^, affecting more than three out of 4 individuals with diabetes mellitus of more than 15 years duration^3^. DR is classified into nonproliferative and proliferative stages. Nonproliferative diabetic retinopathy (NPDR) involves progressive intraretinal microvascular alterations that can evolve to a more advanced proliferative stage defined by extraretinal neovascularization impacting both central and peripheral vision. In 1993, the Diabetes Control and Complications Trial (DCCT)^4^ demonstrated that intensive metabolic control reduces time-averaged blood glucose values (measured as hemoglobin A1c), and also the incidence and progression of DR. Treatment for DR relies almost exclusively on managing the metabolic dysregulation of diabetes until the severity of vascular lesions, such as clinically significant macular edema or proliferative diabetic retinopathy, warrant prompt treatment. Early detection and timely management of DR can prevent vision loss. In particular, identifying the retinal dysfunction at the early stage of DR before clinical signs are apparent could result in earlier medical intervention and better visual outcomes for patients. Microaneurysms are usually the first visible sign of DR. However, microaneurysms do not affect vision and often go unnoticed as a result. Acellular capillaries, devoid of epithelial cells and pericytes, appear adjacent to the clusters of microaneurysms^5^. Regions of acellular capillaries in histologic sections correspond to areas of capillary non-perfusion visualized by ancillary ocular imaging^6^. Thus, imaging modalities capable of visualizing changes in retinal microvascular morphology, such as capillary dropout or non-perfusion, are mostly desired for detecting early DR pathology. Dye-based retinal angiography methods, such as fluorescein angiography and indocyanine green angiography, are invasive, costly, time-consuming, and thus not routinely performed in patients with early-stage DR. Optical coherence tomography angiography (OCTA) has emerged as a non-invasive, three-dimensional technique for visualizing the microvasculature of the retina in different layers at micron-scale resolution^7–10^.

The core principle of OCTA is the detection of OCT signal changes over time, caused by the intravascular motion of blood cells. OCTA imaging in this study was performed with Spectralis OCTA Module using a full-spectrum probabilistic approach. It is worth noting that various OCTA algorithms have been established by several manufacturers. Each OCTA system may differ with regards to the optical source, acquisition speed, scan area, retinal layer segmentation, among others. Such variances in each device may make output images different from one another, which may result in different clinical diagnostic interpretations^11–13^. Quantitative analyses of retinal capillary dropout using OCTA imaging could provide promising biomarkers of early-stage DR. Vessel density or non-perfusion areas (a compliment of vessel density) has been used as a quantitative index to characterize DR-associated changes in retinal microvasculature, revealing that the total non-perfused area is significantly higher in DR subjects compared to normal controls^14–17^, and that decreasing vessel density associates with worsening DR^18,19^. The importance of quantitative assessment of retinal microvasculature in the context of early detection of DR would lie in its distinguishing power for mild NPDR. Vessel density alone may not effectively separate mild DR from healthy subjects. Kim et al.showed that no statistically significant difference of vessel density at the deep retinal layer and full layer (non-segmented) was observed between healthy and mild NPDR^18^. More quantitative imaging tools using OCTA would definitely contribute to more accurate detection of early-stage DR. The goal of this study is to develop and apply a novel quantitative approach to capture local variations in the retinal microvascular orientation as a biomarker-level predictor of DR using advanced OCTA image analysis.

The orientation of tube-like structures has been of great interest to researchers in materials science. For instance, the orientation of individual fibers of steel-fiber reinforced cementitious composites plays an important role in the mechanical properties of the material^20,21^. Hessian matrix-based analysis offers a useful tool for quantification of tube-like structure on digital images. The matrix of the second-order partial derivative of local structure in an image is termed as the Hessian matrix. In computer vision, early approaches to ridge and valleys identification were proposed by Haralick in 1983 utilizing the second directional derivative^22^. Frangi et al.applied eigenvalue (eigenvector) analysis of the Hessian matrix to enhance the vessel structure in angiography image^23^. Specifically, the eigenvector corresponding to the smallest eigenvalue in absolute value was used to estimate the longitudinal direction of the vessel. Geometrical structure measures calculated from eigenvalues examined the likelihood of the vessel presence in the context of developing a vessel enhancement filter. This vessel filter has been widely used in angiography to improve visualization of human vasculature^24^, and served as a preprocessing procedure for the segmentation of blood vessels^25^.

Although the detection of vessel orientation is the intermediate step in the vessel enhancement process, a comprehensive framework for quantification of vessel orientation has never been established in retinal vasculature images. In this study, an operator combining the second derivative and Gaussian multiscale convolution is applied to tune the vesselness filter response that incorporates the eigenvalues, with the objective of enhancing the vessel structure and identifying the retinal vessel width and orientation using OCTA images. This pilot study aims to extract quantitative metrics from the pattern of retinal vascular orientation, namely, vessel preferred orientation, vessel anisotropy and vessel area, to characterize DR-associated changes in retinal microvasculature.

## Methods

### Second derivative

**Figure 1:**
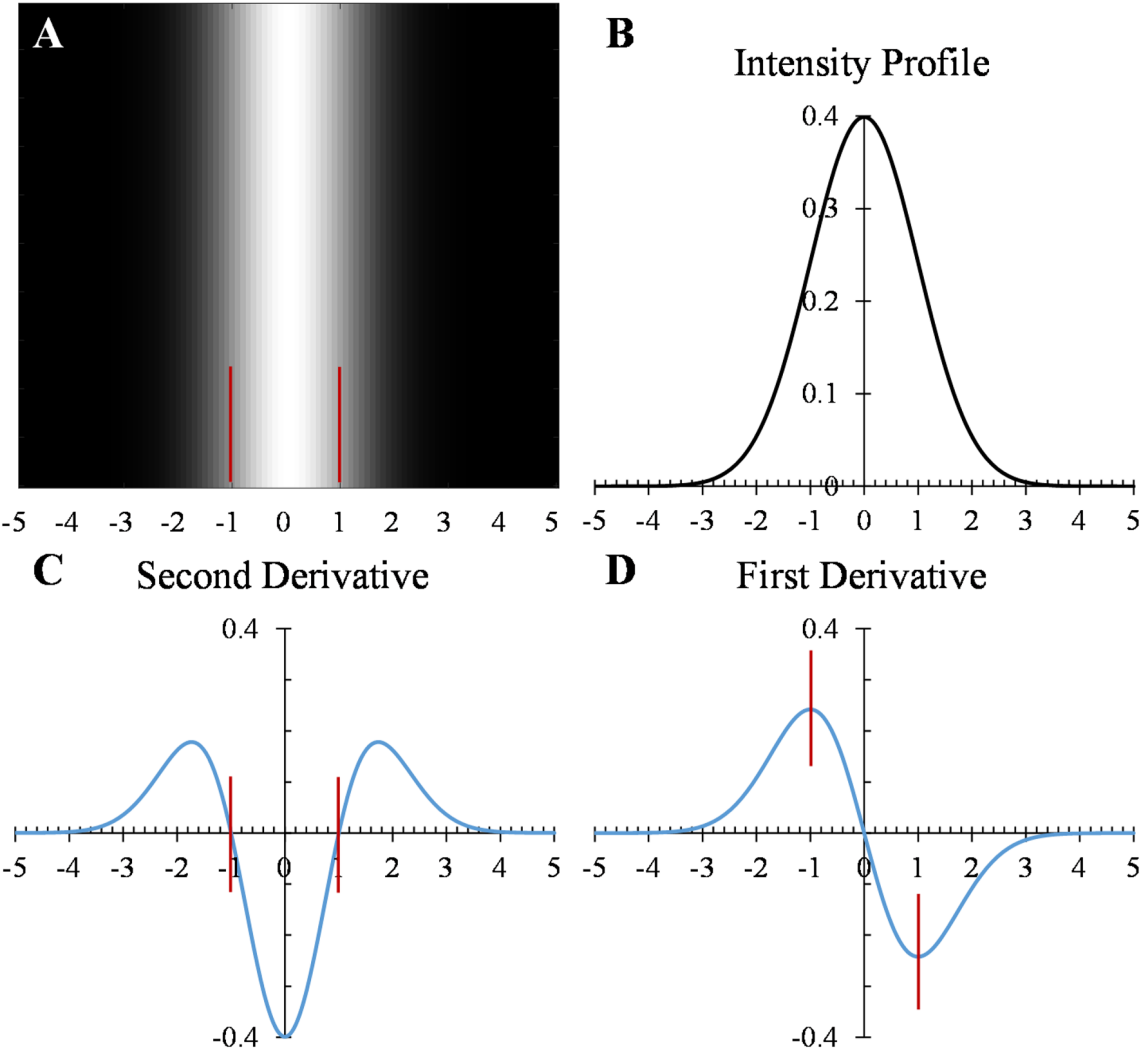
**(A)** Gray-scale image of a tube-like structure with **(B)** an intensity profile of 2-dimensional Gaussian with standard deviation s = 1. Zero-crossings of the second derivative **(C)** correspond to the local maxima in the first derivative **(D)**.

In computer science, the second derivative of the intensity in a gray-scale image can be used as an edge-detection operator. Zero-crossings of the second derivative for a continuous intensity profile correspond to the local maxima in the gradient of the image (first derivative) (Fig. 1). For a vessel modeled as a tube with a 2-dimensional Gaussian profile with standard deviation $s=1$, as specified by ${I}_{0}=\frac{1}{2\pi {s}^{2}}\mathrm{exp}(\frac{-{x}^{2}}{2{s}^{2}})$ (Fig. 1B), the Hessian matrix can be expressed as

1 $${H}_{0}=\left[\begin{array}{cc}\frac{{\partial }^{2}{I}_{0}}{\partial {x}^{2}}& \frac{{\partial }^{2}{I}_{0}}{\partial x\partial y}\\ \frac{{\partial }^{2}{I}_{0}}{\partial x\partial y}& \frac{{\partial }^{2}{I}_{0}}{\partial {y}^{2}}\end{array}\right]=\left[\begin{array}{cc}({x}^{2}-1){I}_{0}& 0\\ 0& 0\end{array}\right]$$

Eigenvectors (${{\varvec{v}}}_{1}$, ${{\varvec{v}}}_{2}$) and eigenvalues (${\lambda }_{1}$, ${\lambda }_{2}$; ${|\lambda }_{1}|<{|\lambda }_{2}|$) of the Hessian matrix ${H}_{0}$ are

$${\lambda }_{1}=0; { {\varvec{v}}}_{1}=\left(\mathrm{0,1}\right);$$

2 $${\lambda }_{2}=\left({x}^{2}-1\right){I}_{0}; {{\varvec{v}}}_{2}=\left(\mathrm{1,0}\right).$$

The orientation of the vessel ${I}_{0}$ is along the y-direction, as shown in Fig. 1A, and is the same as the eigenvector corresponding to the smallest eigenvalue in magnitude, i.e., ${{\varvec{v}}}_{1}$.

For an angiography image, the intensity takes a general form $I ({\varvec{t}})$ which can be approximated by its Taylor expansion in the neighborhood of a point ${{\varvec{t}}}_{0}$ up to the second order to analyze the local structure,

3 $$I\left({\varvec{t}}\right)\approx I\left({{\varvec{t}}}_{0}\right)+\Delta {{\varvec{t}}}^{T}\nabla I\left({{\varvec{t}}}_{0}\right)+\frac{1}{2}\Delta {{\varvec{t}}}^{T}H\left(I\left({{\varvec{t}}}_{0}\right)\right)\Delta {\varvec{t}}$$

where $\Delta {\varvec{t}}={\varvec{t}}-{{\varvec{t}}}_{0}$, $\nabla I\left({{\varvec{t}}}_{0}\right)$ and $H\left(I\left({{\varvec{t}}}_{0}\right)\right)$ are the gradient vector and Hessian matrix of the image $I ({\varvec{t}})$ computed at the point ${{\varvec{t}}}_{0}$, respectively. The third term in Eq. (3) gives the second-order directional derivatives,

4 $$\Delta {{\varvec{t}}}^{T}H\left(I\left({{\varvec{t}}}_{0}\right)\right)\Delta {\varvec{t}}=\left(\frac{\partial }{{\partial {\varvec{t}}}_{0}}\right)\left(\frac{\partial }{{\partial {\varvec{t}}}_{0}}\right)I\left({{\varvec{t}}}_{0}\right)$$

As it has been demonstrated with Eq. (1, 2), with an ideal continuous intensity profile for vessel modeling, eigenvalue and eigenvector analysis of the Hessian matrix can reveal the vessel orientation. Eigenvector defines the direction in which it essentially just get scaled up by the linear transformation,

5 $$H\left(I\left({{\varvec{t}}}_{0}\right)\right) {\varvec{v}}=\lambda {\varvec{v}}$$

and it can be stated equivalently as

6 $${{\varvec{v}}}^{T}H\left(I\left({{\varvec{t}}}_{0}\right)\right) {\varvec{v}}=\lambda$$

The similarity between Eq. (4) and Eq. (6) in terms of composition discloses the association of eigenvalue and the second-order structure of the image. Two orthonormal directions are mapped by the Hessian matrix onto the eigenvalues. A circle neighborhood centered at ${{\varvec{t}}}_{0}$ is mapped by the Hessian matrix onto the second-order structure of the image. The eigenvalues extracted from the Hessian matrix describe the strength of the grey-scale variation in all directions for the pixel of interest. The eigenvector, of the smallest eigenvalue (by absolute value) corresponding to the smallest variation in those grey-scale values, delineates the orientation of the vessel in the angiography image at a specific pixel.

### Multiscale convolution

Multi-scale analysis is imperative to detect the vessels with various widths in the angiography image. When incorporating the scale $\sigma$, linear scale space theory is applied^26^ to ensure the well-posed properties of the differential operator of $I$, such as the gradient vector and Hessian matrix. In this framework differentiation is calculated by a convolution with derivatives of Gaussians:

7 $$\frac{\partial }{\partial {\varvec{t}}}I\left({\varvec{t}},\sigma \right)=I\left({\varvec{t}}\right)*\frac{\partial }{\partial t}G\left({\varvec{t}},\sigma \right)$$

where $*$ denotes the convolution, and a Gaussian kernel of width $\sigma$ is given by

8 $$G\left({\varvec{t}},\sigma \right)=\frac{1}{2\pi {\sigma }^{2}}\mathrm{exp}(\frac{-{||{\varvec{t}}||}^{2}}{2{\sigma }^{2}})$$

where ${||{\varvec{t}}||}^{2}$ is the squared length of vector ${\varvec{t}}$, i.e., ${x}^{2}+{y}^{2}$. The partial second derivative of $I\left({\varvec{t}},\sigma \right)$ in the Hessian matrix can be replaced by the partial second derivative of Gaussian, for example,

9 $${I}_{xx}\left({\varvec{t}},\sigma \right)=I\left({\varvec{t}}\right)*\frac{{\partial }^{2}}{{\partial }^{2}x}G\left({\varvec{t}},\sigma \right)$$

Convolving the image with a Gaussian function can smooth out the image background noise and enhance image vessel structures. The eigenvectors and eigenvalues of the Hessian matrix depends on the scale $\sigma$ of the Gaussian, and therefore are denoted as, ${{\varvec{v}}}_{i}({\varvec{t}},\sigma )$ and ${\lambda }_{i}({\varvec{t}},\sigma )$, respectively (i = 1,2; |${\lambda }_{1}$| <|${\lambda }_{2}$|). The condition of a line can be regarded as ${\lambda }_{1}\approx 0$ (for an ideal line, ${\lambda }_{1}=0$ ), thus the ratio of eigenvalues has been suggested as a similarity measure of a line structure^27,28^,

10 $$R=\frac{{|\lambda }_{1}\left({\varvec{t}},\sigma \right)|}{{|\lambda }_{2}\left({\varvec{t}},\sigma \right)|}$$

In addition to geometric measure for vessels, another important measure is defined to distinguish the vessel from the background noise, termed as a structure-ness measure^23^:

11 $$S=\sqrt{{\lambda }_{1}^{2}\left({\varvec{t}},\sigma \right)+{\lambda }_{2}^{2}\left({\varvec{t}},\sigma \right)}$$

$S$ will be low for the background when there is no presence of the vessel structure as the eigenvalues will be small due to the lack of contrast. With these two measures, $R$ and $S$, a filter response function is defined to detect the vessels with different widths,

12 $$\rho \left({\varvec{t}},\sigma \right)=\mathrm{exp}\left(-\frac{{R}^{2}}{2{\beta }^{2}}\right)\left(1-\mathrm{exp}\left(-\frac{{S}^{2}}{{2\gamma }^{2}}\right)\right)$$

where $\beta$ and $\gamma$ are suppression index. This filter is examined at different scales in the range of ${\sigma }_{min}\le \sigma \le {\sigma }_{max}$, which covers the range of vessel width in the angiography image. The strongest response indicates the identification of vessel width at a specific pixel,

13 $$\rho \left({\varvec{t}}\right)=\underset{{\sigma }_{\mathit{min}}\le \sigma \le {\sigma }_{\mathit{max}}}{\mathrm{max}}\rho \left({\varvec{t}},\sigma \right)$$

The filter response will be maximum when the scale matches the width of the vessel, ${\sigma }_{0}$. The vessel orientation is estimated as ${{\varvec{v}}}_{1}({\varvec{t}},{\sigma }_{0})$, i,e, the eigenvector corresponding to the smallest eigenvalue in magnitude ${\lambda }_{1}({\varvec{t}},{\sigma }_{0})$. Overall, this vesselness filter allows enhancing the vessel-background segmentation and detecting the vessel width and orientation simultaneously. The enhancement quality and efficiency were regulated by four filter parameters, i.e., scale range [${\sigma }_{min}, {\sigma }_{max}$], and suppression index, $\beta$ and $\gamma$. They were empirically determined by approximating the size (in pixels) of the vessel width and evaluating the noise and background suppression.

### Pattern of retinal vascular orientation: preferred orientation, vessel anisotropy and vessel area

**Figure 2:**
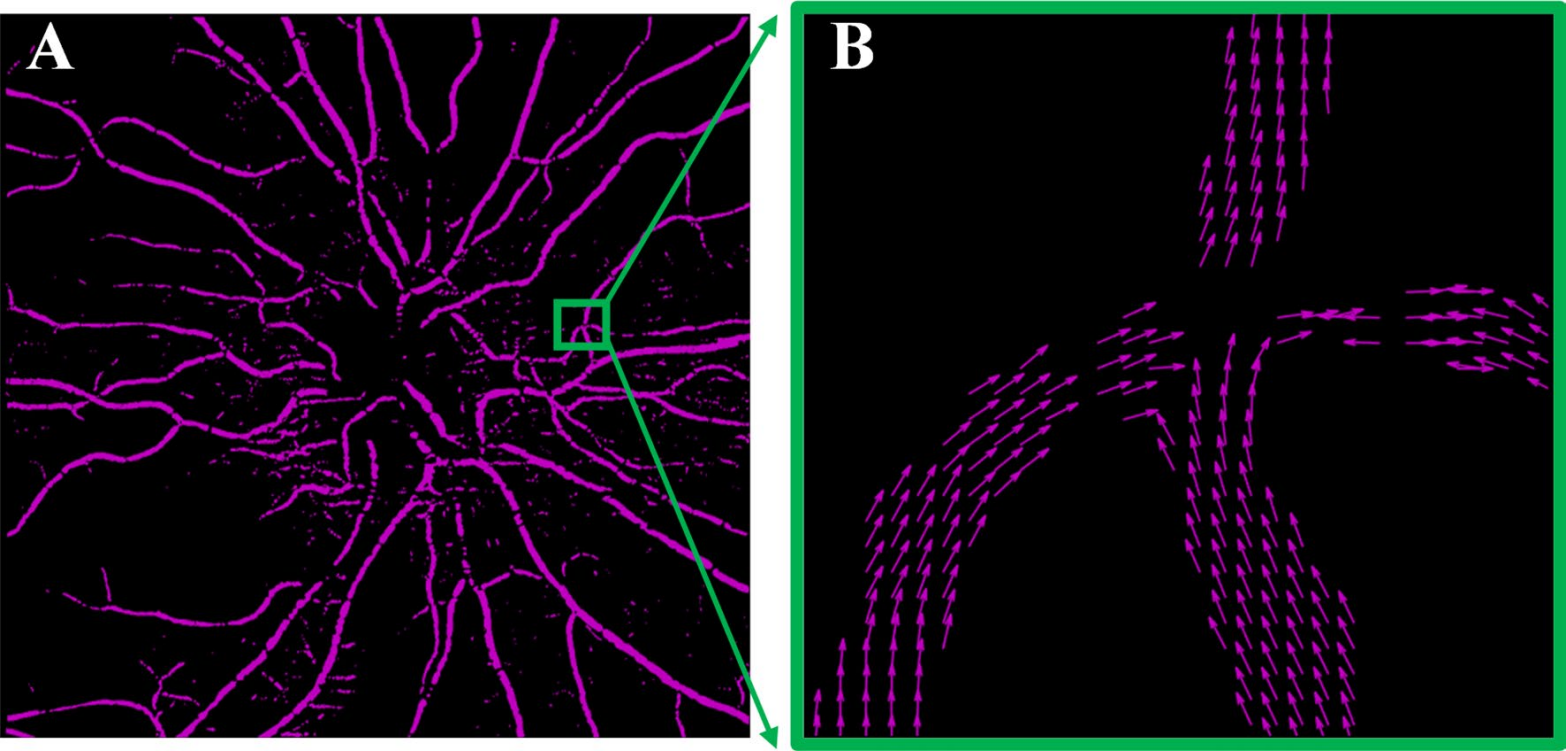
Illustration of vessel orientation extraction from OCTA image. **(A)** Vessel orientations were calculated by the Hessian matrix-based algorithm. **(B)** Enlargement of **(A)**: the arrows indicate the vessel directions/orientations. Note the algorithm is demonstrated on large vessels for illustration.

**Figure 3:**
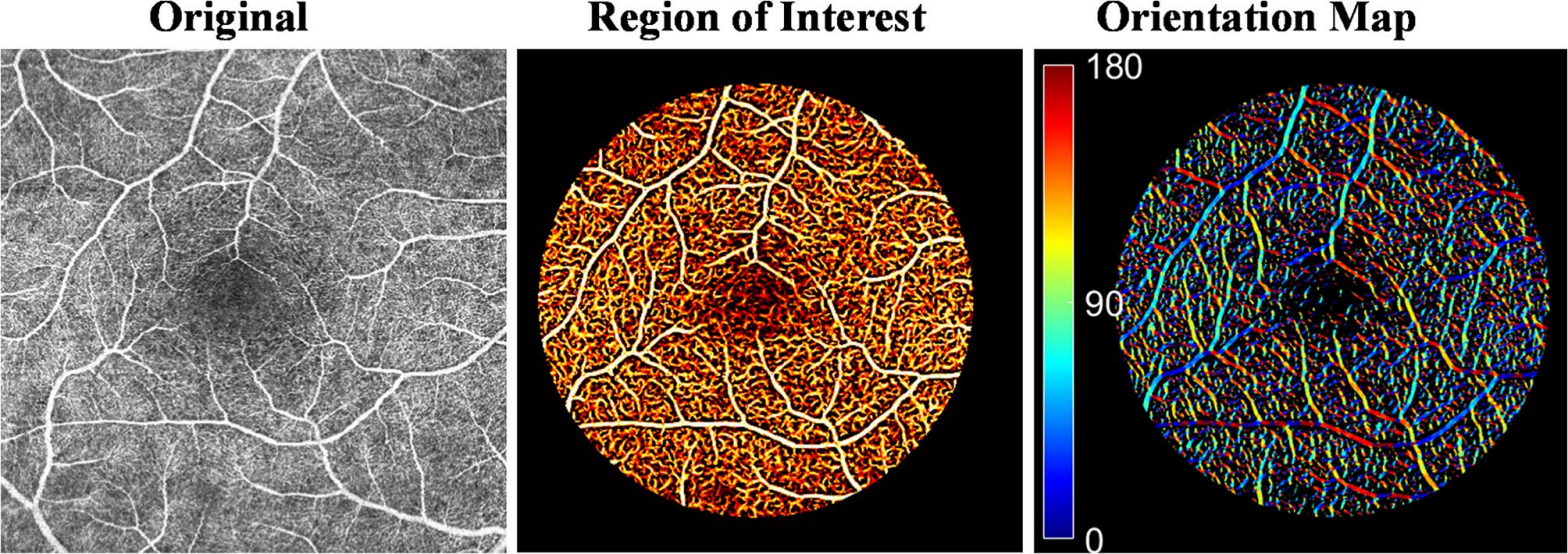
Visualization of retinal microvascular orientation in the region of interest after vesselness filter and binary filter. Dark blue and red indicate 0º and 180º for those horizontal vessels and green indicates 90º for those vertical vessels. The image processing was operated on the full-thickness (non-segmented) en face OCTA image.

Localized changes in retinal microvascular orientation have not been previously quantified from OCTA images. Figure 2A illustrates the vessel orientation extraction from a representative OCTA image. Note that the orientation of the retinal vessel can be identified at each pixel, denoted by the arrows (Fig. 2B). In addition to the vesselness filter, the binary filter was applied prior to the Hessian matrix-based method to extract the vessel orientation in the region of interest (ROI). Binary filter with a fixed threshold was limited by the fact that the noise level could vary among scans and even within the same scan due to deviations in the OCT reflectance signal^29,30^. In contrast, we created a binary vessel mask with a globally determined threshold using Otsu’s method^31^, which chooses the threshold value to minimize the intra-class variance of the black and white pixels in the image. Color maps were generated to visualize the local vessel orientation in the ROI (Fig. 3).

**Figure 4:**
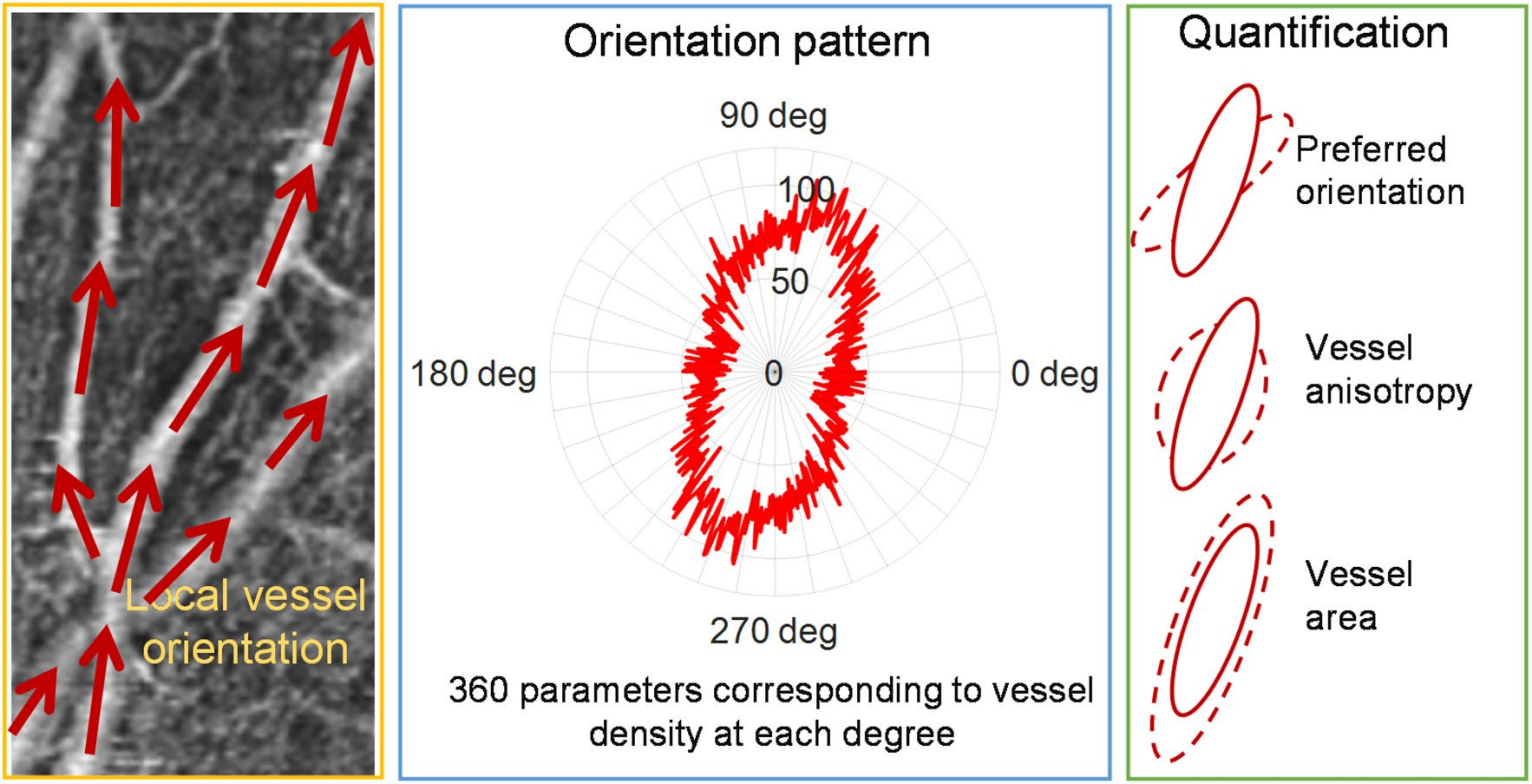
Quantification of vascular orientation pattern using preferred orientation, vessel anisotropy, and vessel area. The orientation pattern (middle) for the specific ROI (left) depicts a roughly elliptical shape with a major axis and a minor axis. The preferred orientation is identified by the angle of the major axis. The ratio of major axis length and minor axis length is defined as vessel anisotropy. The vessel area is defined as the area of the shape. Examples of preferred orientation, vessel anisotropy, and vessel area, are illustrated by the dashed ellipse relative to the solid ellipse (right).

A polar plot of orientation distribution was generated to show the probability of vessel at each angle from 0 to 360 degrees. This orientation distribution curve exhibits the unique pattern of vasculature organization in the selected ROI. Quantitative measures of the vessel orientation pattern can be achieved by analyzing the polar plot region encompassed by the orientation distribution curve, including preferred orientation, vessel anisotropy, and vessel area. As shown in Fig. 4, the orientation pattern for the specific ROI (middle) depicts a roughly elliptical shape with a major axis and a minor axis. The preferred orientation with the unit of degree is identified by the angle of the major axis. The ratio of major axis length and minor axis length is defined as the unitless vessel anisotropy. The vessel area with the unit of pixel^2^ is defined as the area of the shape or the number of square pixels that covers the closed orientation distribution curve. These three quantitative metrics are independent of each other (Fig. 4).

Descriptive statistics including mean, standard deviation, and range were reported for preferred orientation, vessel anisotropy, and vessel area in each sector of the OCTA image in the healthy and DR cohorts. The statistical analysis was performed by using linear mixed-effect models for analyzing retinal vascular patterns in different sectors of the OCTA image. Non-parametric Mann–Whitney U test (also called Wilcoxon rank sum test) was used to compare the data between DR cases and healthy controls. The correlation between vessel area quantified from the pattern of retinal vascular orientation in the current study and traditionally reported vessel density^24^ was evaluated by Pearson correlations. A probability (p) value of 0.05 or less was considered to be statistically significant. All data analysis was conducted by using SAS software (V9.4; SAS Institute Inc., Cary, NC, USA, https://www.sas.com/).

### Subject participants

All experiments were performed in adherence to the tenets of the Declaration of Helsinki and informed consent was obtained from all participants. This study was approved by the Institutional Review Board of The Ohio State University. This was a retrospective, cross-sectional, observational study of healthy controls and DR cases. Inclusion criteria for both DR subjects and healthy controls were age 18 years or greater, absence of prior intraocular surgery (except for cataract), corneal pathology and retinal pathology, ability to comprehend, agree, and sign the subject informed consent form, and willingness to comply with the prescribed schedule at the time of enrollment. The disease severity level of the included DR subjects was ranked as mild and moderate (without current evidence of macular edema) based on the modified Airlie House/Early Treatment Diabetic Retinopathy Study (ETDRS). Exclusion criteria for participants included any history of ocular injury and ocular diseases, such as age-related macular degeneration, glaucoma, ocular hypertension, keratoconus, or proliferative diabetic retinopathy. Participants who had a diagnosis of retinal detachment, retinal tear, retinal degeneration, or retinal hole were excluded. Participants were excluded if they were pregnant, less than 12 weeks postpartum, or less than 12 weeks since the last breastfeeding activity. Further, participants with spherical equivalent refraction < -6 diopters or more than + 6 diopters were also excluded.

For all participants, OCTA images were acquired with the Spectralis OCTA module (Heidelberg Engineering, Heidelberg, Germany). OCT volume scans centered at the macula were taken with dimensions of 6 × 6 × 2 mm consisting of 512 clusters of B-scans with a distance of 11 µm between B-scans. Active eye-tracking (TruTrack) technology was used to correct for displacements by re-acquisition of OCT images at the correct retinal location in real-time. Any images with significant artifactual components due to blockage of OCT signal by floaters and eyelashes, residual motion artifacts, or other artifacts, were excluded from the study to avoid confounding of quantitative analysis. A circular area centered at the macula with a diameter of 5 mm was used (instead of the entire 6 × 6 mm^2^) for the aforementioned image processing to reduce the effects of the artifacts at the edge of the scan.

## Results

Forty-one subjects were imaged in this study for the quantitative assessment of retinal microvasculature, including 34 healthy controls (age: 21–59 years) and 7 DR cases (age: 24–65 years). Subjects within the healthy and DR cohorts were not significantly different in age (p = 0.077). Only one eye (right eye) per subject was included in the analysis.

### Intra-subject sectoral differences in retinal microvascular orientation patterns

**Figure 5:**
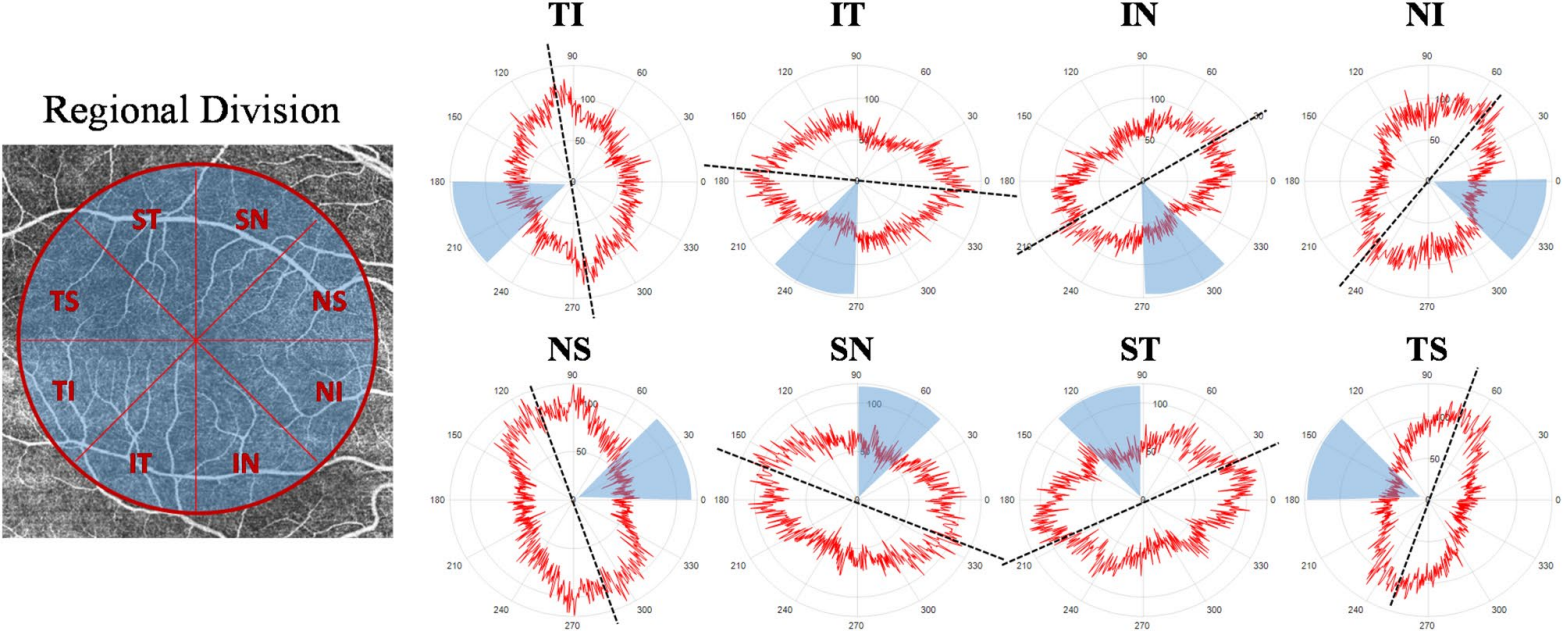
Sectoral vascular pattern with preferred vessel orientation (dashed line) unaligned with sector axis. Eight 45º sectors were divided from a circular disk centered at the macula and each sector was defined as the region of interest for the quantitative assessment of retinal vascular orientation pattern. *N* nasal, *S* superior, *T* temporal, *I* inferior.

**Figure 6:**
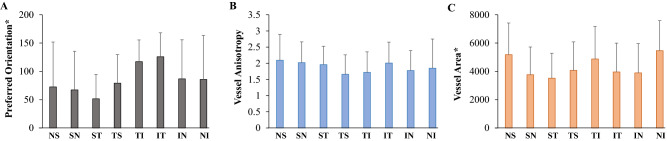
Sectoral difference in retinal microvascular pattern in healthy subjects (n = 34). Significant sectoral differences were observed in preferred orientation (p < 0.0001) and vessel area (p < 0.0001), whereas vessel anisotropy did not show a significant difference among the 8 sectors (p = 0.054).

Our quantitative analysis defines the ROIs as equal-area sectors. Each 45º sector of the circular disk centered at the macular region was defined as the new ROI, namely NS, SN, ST, TS, TI, IT, IN, and NI (N = nasal, S = superior, T = temporal, I = inferior). Sectoral preferred orientations of retinal microvasculature were shown to vary within the same eye and are unaligned with their sector axis as shown in Fig. 5. Vessel orientation pattern for each sectoral ROI was quantified, including preferred vessel orientation, vessel anisotropy and vessel area, and compared among the 8 sectors in the healthy subjects (n = 34). Linear mixed model analysis was performed to account for the association of the quantification at different sectors from the same image. Intra-subject sectoral variations were demonstrated in the bar graph for preferred vessel orientation (Fig. 6A), vessel anisotropy (Fig. 6B), and vessel area (Fig. 6C). In the healthy cohort, significant differences were observed among the 8 sectors in preferred vessel orientation (p < 0.0001) and vessel area (p < 0.0001), while no significant sectoral difference was observed in vessel anisotropy (p = 0.054).

### Differential retinal microvascular orientation patterns between healthy and DR cohorts

In the SN and ST sector, the average preferred vessel orientation of healthy subjects was similar to that of DR subjects (SN: 67.2° ± 68.4° for healthy vs 62.2° ± 77.1° for DR; ST: 51.7° ± 43.1° for healthy vs 49.4° ± 31.2° for DR). The largest difference in preferred orientation between healthy and DR was observed in the IN sector (86.8° ± 69.1° for healthy vs 157.2° ± 29.4° for DR, p = 0.005). It is worth noting that due to the head–tail nature of the preferred orientation, i.e., 0° and 180° define the same line direction, when the standard deviation in a certain sector for healthy or DR subjects is greater than 45°, it is very likely that the preferred orientation for some subjects falls into the first quadrant closer to 0° and some fall into the second quadrant closer to 180° (see Table 1). For the vessel anisotropy, no significant difference was observed in any of the eight sectors between healthy and DR cohorts. The average vessel area in DR is smaller than healthy subjects in the TI and IT sectors (TI: 4879 ± 2297 pixel^2^ for healthy vs 2277 ± 1464 pixel^2^ for DR, p = 0.004; IT: 3967 ± 2025 pixel^2^ for healthy vs 2420 ± 1592 pixel^2^ for DR, p = 0.046).

**Table 1:** Quantification of retinal microvascular pattern for 34 healthy and 7 DR subjects, including the average value, standard deviation and range for preferred orientation, vessel anisotropy and vessel area in each sector.

	Healthy (n = 34)			Diabetic retinopathy (n = 7)		
	Mean ± SD	Minimum	Maximum	Mean ± SD	Minimum	Maximum
**Preferred orientation (°)**						
NS	72.5 ± 79.2	0.5	178.7	38.3 ± 59.6	3.9	168.6
SN	67.2 ± 68.4	1.5	175.2	62.2 ± 77.1	2.1	178.2
ST	51.7 ± 43.0	1.3	177.2	49.4 ± 31.2	15.8	105.2
TS	79.3 ± 50.3	0.4	177.9	92.4 ± 53.5	2.7	158.7
TI	117.2 ± 38.3	5.2	178.7	86.7 ± 60.7	1.6	158.8
IT	125.9 ± 42.3	5.9	176.2	137.1 ± 22.0	103.5	167.9
IN*	86.8 ± 69.1	1.4	174.8	157.2 ± 29.4	109.6	179.5
NI	85.8 ± 77.6	0.6	179.8	145.0 ± 62.8	3.3	179.5
**Vessel anisotropy**						
NS	2.1 ± 0.8	1.2	4.5	1.9 ± 0.3	1.5	2.4
SN	2.0 ± 0.6	1.1	3.7	2.5 ± 1.4	1.5	5.1
ST	2.0 ± 0.6	1.3	4.1	1.7 ± 0.5	1.2	2.4
TS	1.7 ± 0.6	1.0	3.8	1.8 ± 0.7	1.1	2.8
TI	1.7 ± 0.6	1.0	3.7	2.4 ± 0.9	1.1	3.7
IT	2.0 ± 0.6	1.0	3.6	2.0 ± 0.8	1.3	3.4
IN	1.8 ± 0.6	1.0	4.0	1.9 ± 0.6	1.1	2.6
NI	1.8 ± 0.9	1.2	5.2	1.9 ± 0.5	1.2	2.6
**Vessel area (pixel**^**2**^**)**						
NS	5182 ± 2240	2226	10,824	4988 ± 1922	1885	7470
SN	3769 ± 1952	1498	9884	3433 ± 1183	1815	5061
ST	3525 ± 1754	1273	8832	2631 ± 1281	854	3903
TS	4072 ± 2012	1355	11,171	2966 ± 1762	1040	5403
TI*	4879 ± 2297	1379	10,486	2277 ± 1464	652	5160
IT*	3967 ± 2025	1274	9331	2420 ± 1592	736	5063
IN	3896 ± 2063	842	10,028	2345 ± 1835	455	4972
NI	5461 ± 2125	2072	11,353	4735 ± 3001	1145	9605

Asterisk * indicates statistically significant (p < 0.05) between healthy and DR cohorts using Mann–Whitney U test.

*SD* standard deviation.

Table 1 lists the quantification of retinal microvascular patterns for healthy and DR subjects including the average, standard deviation, and the range for each metric in each sector. Asterisk indicates statistical significance at the level of 0.05 using the non-parametric Mann–Whitney U test for the comparison of DR and healthy subjects. Although this study does not focus on hypothesis comparison due to the small sample size especially in the DR cohort, these asterisk marked sectors (e.g., IN for preferred orientation, TI and IT for vessel area) could serve as important preliminary data for the design of future studies.

### Comparison between vascular orientation pattern and vessel density

**Figure 7:**
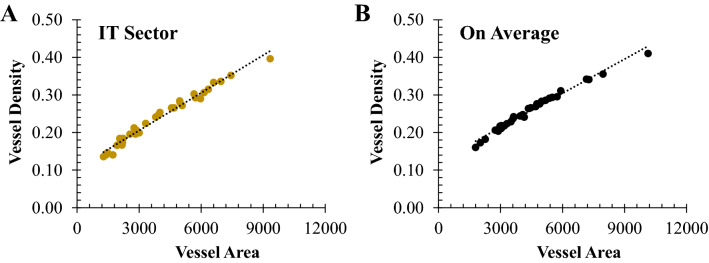
Scatterplot of the relationship between vessel area and vessel density in **(A)** the IT sector and **(B)** the average of 8 sectors in healthy subjects. The vessel area quantified from the retinal vascular orientation pattern was strongly correlated with the vessel density with Pearson R = 0.99 for both IT sector and on average (p < 0.0001, n = 34).

Vessel density was calculated as the ratio of the vasculature to the total image area in the ROI in the binary vessel map^24^. Theoretically, the vessel area quantified from our vascular orientation pattern shares the same concept as the vessel density which sums up the total pixels of vessels in the ROI. We have compared the vessel area and the vessel density for 34 healthy subjects in each sector. The vessel density and vessel area were strongly correlated with Pearson R > 0.97 at every sector (p < 0.0001 for all 8 sectors). In addition, since our quantitative analysis defines the ROIs as equal-area sectors, the mean value of vessel density in the 8 sectors represents the overall density in the circular disk centered at the macula. The averaged vessel density and averaged vessel area in the eight sectors were also strongly correlated (R = 0.99, p < 0.0001). Figure 7 provides scatterplots of the relationship between vessel area and vessel density in the IT sector and on average. Our quantitative analysis for vascular orientation pattern not only provides vessel area, but also preferred orientation and vessel anisotropy simultaneously, demonstrating its advantage as a quantitative tool over vessel density alone.

## Discussion

The pathologic mechanism of DR is directly related to the underlying hyperglycemia associated with diabetes. The best way to prevent or delay the progression of DR is early and aggressive control of one’s blood sugar. Hemoglobin A1c remains the only confirmed systemic prognostic biomarker of DR progression^32^. However, the fact that diabetics with appropriate glycemic control still develop vision loss exemplifies the need for additional markers of DR onset and progression. In the early stage of DR, patients are generally asymptomatic. Ophthalmic imaging acquired from the patient’s routine eye examination could offer a way to identify and track the cumulative damage from hyperglycemia. Thus, advanced image-based quantification may have valuable predictive information and provide more efficient management of the patient with DR. Several quantitative metrics using en face OCTA images have been developed to objectively characterize the retinal vessels with the objective of detecting or staging DR. Vessel density over a desired region of interest is the most common quantitative assessment made with OCTA^18,19^. Although vessel density measurements showed statistically significant differences between the DR and healthy eyes, it had limited sensitivity to detect DR at early stage^33^. Measurements of the vessel density using OCTA showed relatively good repeatability for various retinal diseases including diabetic macular edema, retinal vein occlusion with macular edema, epiretinal membrane, and wet age-related macular degeneration^34^. However, the decrease in vessel density was not only observed in DR, but also in other retinal disease^35,36^, suggesting poor specificity for DR detection using vessel density. Noting the limitation of vessel density as a quantitative metric to characterize DR-associated changes in retinal microvasculature, automatic segmentation algorithms have been developed to extract vascular features such as geometric perfusion deficits^37^, foveal avascular zone^38^, inter-capillary area^17^, and fractal dimension^39^. To the best of our knowledge localized changes in microvascular orientation have never been quantified from OCTA images, which has the potential to detect disease at the early stage with enhanced sensitivity and specificity. In this study, we developed a novel quantitative approach to delineate the pattern of retinal vascular orientation from OCTA images which generated three quantitative metrics. First, Gaussian multi-scale convolution was combined with the second derivative in an attempt to tune the vesselness filter response to the specific vessel width and orientation. Then with the identification of vessel orientation at each pixel, the pixels at a certain angle ranging from 0 to 360 degrees were integrated, which yielded the orientation pattern in the desired ROI. Preferred vessel orientation, vessel anisotropy, and vessel area were defined to quantify the orientation pattern, reducing 360 descriptive metrics to a manageable three metrics. Our results have elucidated that this novel quantitative approach is more advanced than the traditionally reported vessel density in two aspects:

(1) Extra metrics were achieved by quantifying the vessel orientation pattern beyond a single quantitative analysis of vessel density. The vessel area characterized from the vascular orientation pattern was shown to be strongly correlated with the traditionally reported vessel density. Quantification of vessel preferred orientation and anisotropy that were characterized from the vascular orientation pattern simultaneously along with vessel area could provide additional information about the retinal vasculature. Thus, our vessel orientation-based quantitative assessment for retinal microvasculature hold promise for DR detection at the earliest stage.

(2) Sectoral analysis for the retinal vasculature may have the potential for the development of a biomarker for the DR disease. For quantitative imaging when the quantification is the mean value of a parametric map within an ROI, the ROI size and shape should be first considered when assessing and interpreting the results. The most common ROI for OCTA image processing is the square of either 3 × 3 mm^2^ or 6 × 6 mm^2^, however, there might be inter-subject variations of the macula location in OCTA image. In this study, a circular area centered at the macula with a diameter of 5 mm (from 6 × 6 OCTA image) was used to allow an anatomically more consistent comparison among subjects, and to reduce the effects of the artifacts at the edge of the scan. Eight 45º sectors of the circular disk were defined as the ROI. Significant sectoral differences were observed in preferred vessel orientation (p < 0.0001) and vessel area (p < 0.0001) in the healthy controls. Further, vessel preferred orientation and vessel area quantified from our vascular orientation pattern also demonstrated a difference between healthy and DR cohorts in certain sectors. Our sectoral analysis therefore may show better performance in identifying focal defects manifested in DR.

This pilot study focused on the method development with initial, proof-of-concept results on the differential retinal microvascular orientation patterns between healthy and DR subjects to demonstrate the feasibility and advantage of this approach. The observed significant differences in the pattern of vascular orientation between DR and healthy such as IN for preferred orientation, TI and IT for vessel area, offered preliminary data for future study design. The quantification in this study focused on the full projection of the OCTA image (summing up all the retina layers in the thickness direction). Quantification of the retinal vascular pattern within different layers is currently under investigation to further analyze the regional difference. The small sample size was the major limitation. With our quantitative method established with pilot data in this study, a follow-up study is ongoing to provide more robust normative data in healthy subjects, and to further investigate the vascular change in DR using our quantitative analysis of vascular orientation pattern with larger sample size. Another application of the retinal microvascular orientation would be to evaluate the tortuosity in vessels recognized in digital fundus images or OCTA images. Tortuosity is one of the first alterations in the retinal vasculature in hypertensive retinopathy. For instance, hypertensive patients have severe vessel tortuosity compared to healthy subjects who exhibit normal/very mild vessel tortuosity. Some approaches for tortuosity measurement have been proposed, but they do not always coincide with ophthalmologist’s perception of vessel tortuosity^40^. Vessel orientation-based tortuosity evaluation warrants further investigation.

In conclusion, our novel quantitative approach using OCTA imaging allows us to map and quantify the retinal microvascular orientation pattern, which in turn holds promise for the early detection of DR-associated retinal vascular abnormalities.
